# The direct impact of derotation techniques (omt-kalternborn-evjenth) on the trunk morphology and the possibility of the autocorrection in girls with AIS – pilot studies

**DOI:** 10.1186/1748-7161-9-S1-O67

**Published:** 2014-12-04

**Authors:** Jacek Durmala, Bartosz Wnuk, Irmina Blicharska

**Affiliations:** 1 Department of Rehabilitation, Medical University of Silesia, Katowice, Poland

## Background

Conservative treatment of idiopathic scoliosis is a difficult and complex therapeutic process, witch not always is finished successfully. It seems that manual therapy may be used as an element in the preparatory phase for active 3D specific exercises. A systematic review of the literature (which is rather poor) performed by Romano and Negrini show, that manual therapy is not support in the treatment of the idiopathic scoliosis.

## Aim

The aim of this study was to assess the direct impact of derotation techniques (Orthopedic Manual Therapy-Kalternborn-Evjenth) on the trunk morphology and on the possibility of the autocorrection in active exercises in girls with adolescent idiopathic scoliosis.

## Design

Prospective, randomized and double blind studies.

## Methods

16 girls (15±2y.) with AIS (DM – mean Cobb Th=26±7° and L=23±6°). The trunk morphology was examined in two standing positions – habitual and corrected in front of mirror (surface topography – position of pelvis in three planes and clinic examination - kyphosis and lordosis by plurimeter) in the morning before and after 10 minutes mobilizations. Active and passive derotational mobilization techniques were used only in the area of lumbar curvature. Derotational mobilization techniques were implemented in according with the concept of OMT Kalternborn-Evjenth in a sitting position. Nonparametric tests were used for statistical analysis.

## Results

Increasing the possibility of the autocorrection of patient’s trunk in visually biofeedback was observed after once performed mobilization technique. Significant differences among the level of the autocorrection of waist were observed. Statistically significant changes were reported only for two parameters (symmetrical level (p<0.04) and depth (p<0.03) of waist).

## Conclusions

The derotational mobilization techniques by Orthopedic Manual Therapy - Kalternborn-Evjenth may be useful in a preparatory phase of the specific active exercises. The topic needs the further investigation.
